# Diagnostic Accuracy and Clinical Utility of Adson’s Test in Detecting Subclavian Artery Compression Associated With Cervical Ribs: A Systematic Review

**DOI:** 10.7759/cureus.94341

**Published:** 2025-10-11

**Authors:** Mazin Osman, Alaaeldin Saad, Sharmila Venkatachalapathi, Shabeh e roshan Ali, Mohamed K Abouelsadat, Kinza Ali, Nimrah Majeed, Kiranjot Kaur, Mashal Mumtaz, Shahid Khan

**Affiliations:** 1 General Surgery, British United Provident Association, Jeddah, SAU; 2 Vascular Surgery, Royal Free Hospital, London, GBR; 3 Internal Medicine, Periyar Government Hospital, Mayiladuthurai, IND; 4 Internal Medicine, Vadamalayan Hospitals Pvt Ltd., Dindigul, IND; 5 General Medicine, Cavan and Monaghan Hospital, Cavan, IRL; 6 Medicine and Surgery, Liaquat University of Medical and Health Sciences, Jamshoro, PAK; 7 Internal Medicine, Cavan General Hospital, Cavan, IRL; 8 Medicine and Surgery, Civil Hospital Karachi, Karachi, PAK; 9 Nephrology, Liaquat National Hospital, Karachi, PAK; 10 General Medicine, Abbasi Shaheed Hospital, Karachi, PAK; 11 Internal Medicine, United States Navy, United States Military, North Chicago, USA; 12 Clinical Research, Arizona State University, Tempe, USA; 13 Internal Medicine, Shri B M Patil Medical College, Bijapur, IND; 14 Internal Medicine, University College of Medicine and Dentistry, University of Lahore, Lahore, PAK; 15 General Surgery, Abbasi Shaheed Hospital, Karachi, PAK

**Keywords:** adson's test, cervical rib, diagnostic accuracy, subclavian artery compression, thoracic outlet syndrome

## Abstract

Thoracic outlet syndrome results from neurovascular compression at the thoracic outlet, with the vascular subtype, often linked to cervical ribs leading to subclavian artery stenosis, aneurysm, or thromboembolism. Adson’s test is a long-standing provocative maneuver used for diagnosis, but its accuracy has been questioned. This systematic review, conducted according to PRISMA 2020 guidelines, searched PubMed, Embase, Scopus, and the Cochrane Library up to August 2025. Studies were included if they involved more than 20 human participants, assessed Adson’s test against imaging or surgical confirmation, and reported diagnostic accuracy outcomes. Risk of bias was evaluated with QUADAS-2 and the Cochrane tool. Out of 276 identified records, six studies with a total of 305 participants met the criteria. Reported sensitivity ranged from 72% to 92%, whereas specificity was consistently low (9-53%). False positives were frequent in healthy volunteers and in patients with overlapping disorders such as carpal tunnel syndrome, but predictive value improved in cases with cervical ribs or when combined with other maneuvers. Overall, Adson’s test demonstrates relatively high sensitivity but poor specificity, limiting its use as a confirmatory diagnostic tool. Its clinical value lies primarily in initial screening and triage, especially in patients with anatomical anomalies such as cervical ribs, while modern imaging remains essential for definitive diagnosis and management.

## Introduction and background

Thoracic outlet syndrome (TOS) refers to a constellation of conditions caused by compression of the neurovascular structures passing through the thoracic outlet. The anatomical space is bounded by the scalene muscles, the first rib, the clavicle, and occasionally a cervical rib. Vascular TOS, a less common but clinically significant subtype, involves compromise of the subclavian artery or vein, leading to ischemic symptoms, arterial stenosis, aneurysm, or even thromboembolic events. Among the structural anomalies, a cervical rib is a known cause of arterial compression, reported in 0.5-1% of the general population but disproportionately represented in symptomatic TOS patients [1]. Such cases underscore the importance of accurate diagnostic tools that can differentiate physiological narrowing from true pathological compression. Adson’s test has long been regarded as a cornerstone provocative maneuver for the diagnosis of TOS. In addition to Adson’s test, the Eden Test, also known as the Military Brace Test or Costoclavicular Syndrome Test, plays an important role in clinical assessment. It is a physical examination used to evaluate TOS, a condition in which nerves or blood vessels are compressed between the clavicle (collarbone) and the first rib. The test involves extension and external rotation of the arm with concurrent head rotation and deep inspiration, aiming to exacerbate narrowing at the interscalene triangle and subclavian artery passage. A positive Eden Test is indicated by a weakened or absent radial pulse or reproduction of the patient’s symptoms.

Historically, the test was believed to demonstrate both arterial and neurologic compromise, but subsequent studies revealed a high prevalence of false positives in asymptomatic individuals [2]. This casts doubt on its discriminative validity and emphasizes the need for contextual interpretation rather than reliance on the test in isolation. Nevertheless, in the presence of anatomical anomalies such as a cervical rib, Adson’s maneuver may amplify diagnostic clues when corroborated by imaging or surgical findings. Modern imaging modalities, including duplex ultrasonography, CT angiography, and MR angiography, provide objective confirmation of vascular compression and are increasingly considered the gold standard in diagnosis [3]. These techniques allow for visualization of dynamic arterial narrowing and collateral circulation, making them essential adjuncts in suspected cervical rib cases. However, such resources may not always be available in initial clinical settings. Consequently, provocative tests like Adson’s remain widely practiced, particularly in outpatient clinics. The challenge lies in interpreting their findings judiciously, balancing their ease of use with their documented limitations.

An integrated approach combining physical maneuvers with imaging increases diagnostic accuracy and helps mitigate misdiagnosis or unnecessary interventions. Systematic reviews and meta-analyses suggest that while Adson’s test demonstrates relatively high sensitivity, its specificity remains consistently low, particularly in healthy volunteers or patients with overlapping conditions such as carpal tunnel syndrome or cervical spondylosis [4]. These findings highlight the need for caution when interpreting a positive result, particularly in populations at risk of misclassification. The presence of a cervical rib alters the anatomical context, potentially increasing the predictive value of the test. Therefore, the primary aim of this systematic review is to evaluate the diagnostic accuracy and clinical utility of Adson’s test in detecting subclavian artery compression specifically associated with cervical ribs. By synthesizing current evidence, this review seeks to clarify the role of Adson’s maneuver in modern clinical practice and its contribution to accurate detection of vascular TOS [5].

## Review

Materials and methods

Search Strategy

This systematic review was conducted in accordance with the Preferred Reporting Items for Systematic Reviews and Meta-Analyses (PRISMA) 2020 guidelines to ensure methodological rigor and transparency [6]. Comprehensive searches were carried out in four major biomedical databases: PubMed, Embase, Scopus, and the Cochrane Library, covering studies published up to August 2025. Only peer-reviewed literature involving human participants and published in English was considered. Two reviewers independently screened all records for eligibility, with disagreements resolved by consensus or referral to a third reviewer. This approach minimized bias and improved the reliability of study selection. A structured search strategy was designed using a combination of Medical Subject Headings (MeSH) and free-text keywords relevant to thoracic outlet syndrome, cervical rib, and diagnostic maneuvers. Search terms included “Adson’s test,” “thoracic outlet syndrome,” “cervical rib,” “subclavian artery compression,” and “provocative maneuvers.” Boolean operators such as “AND” and “OR” were applied to refine the search and optimize both sensitivity and specificity. Filters were applied to restrict results to human studies published in English. This comprehensive strategy was developed to maximize the retrieval of studies relevant to the diagnostic accuracy of Adson’s test.

Eligibility Criteria

Eligibility criteria were defined using a PICO framework [7]. The Population (P) included patients with suspected thoracic outlet syndrome or those with cervical ribs, as well as healthy volunteers serving as controls. The Intervention (I) was Adson’s test performed as a provocative diagnostic maneuver. The Comparator (C) consisted of imaging modalities such as computed tomography angiography (CTA) and duplex ultrasonography, surgical confirmation, or alternative provocative maneuvers. The Outcomes (O) included diagnostic accuracy parameters such as sensitivity, specificity, positive predictive value, and negative predictive value. Studies were included if they were original human research, clearly defined diagnostic criteria, reported measurable accuracy outcomes for Adson’s test, involved sample sizes greater than 20, and were published in English. Studies were excluded if they were case reports, animal research, editorials, or conference abstracts without peer review.

Study Selection

Study selection followed a two-stage process. First, titles and abstracts were screened independently by two reviewers to identify potentially eligible studies. Second, full texts of these studies were assessed for compliance with inclusion and exclusion criteria. Any discrepancies were resolved by consensus or arbitration from a third reviewer. A PRISMA flow diagram was used to illustrate the study selection pathway, ensuring a transparent overview of the screening and exclusion process. This structured approach enhanced the reliability and reproducibility of the review.

Data Extraction

Data extraction was performed systematically using predesigned forms to ensure consistency across included studies. Extracted information included author and year of publication, study population, diagnostic reference standard, outcomes of Adson’s test, and relevant pathophysiological or anatomical findings. To reduce the risk of error, data were collected independently by two reviewers and then cross-verified. Tabulated summaries were created for comparability across studies, and extracted data formed the basis for the synthesis of results.

Risk of Bias Assessment

Risk of bias was assessed using validated tools appropriate to the study type. For diagnostic accuracy studies, the Quality Assessment of Diagnostic Accuracy Studies 2 (QUADAS-2) tool was applied, which evaluates bias across domains such as patient selection, index test, reference standard, and timing [8]. For systematic reviews, the Cochrane Risk of Bias Tool was employed, assessing methodological soundness and heterogeneity of included evidence [9]. Each study was rated as having low, moderate, or high risk of bias, with justifications documented for transparency.

Data Synthesis

Due to significant heterogeneity in study design, populations, and outcome measures, meta-analysis was not feasible. Instead, a narrative synthesis approach was adopted. Accuracy measures of Adson’s test were compared across symptomatic, asymptomatic, and mixed cohorts. Structural factors such as cervical ribs were emphasized when reported.

Results

Study Selection Process

**Figure 1:**
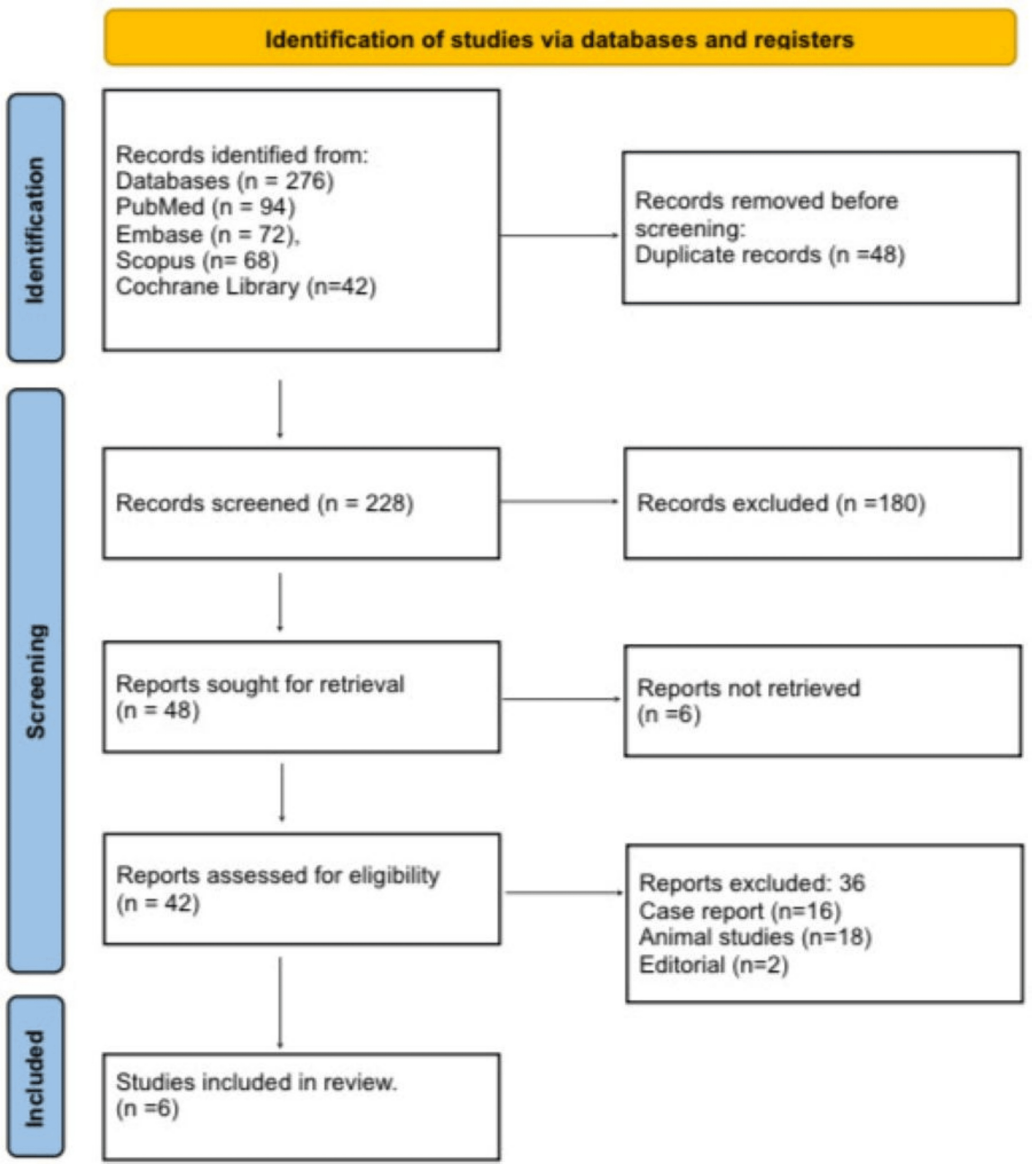
PRISMA 2020 Flow Diagram PRISMA: Preferred Reporting Items for Systematic Reviews and Meta-Analyses

Figure 1 shows that a total of 276 records were identified (PubMed = 94, Embase = 72, Scopus = 68, Cochrane = 42). After removing 48 duplicates, 228 records remained. Title/abstract screening excluded 180 studies (non-relevant, reviews, biomechanics). Full-text review of 42 reports excluded 36 studies (case reports = 16, animal studies = 18, editorials = 2). Six studies were included.

Characteristics of the Selected Studies

Table 1 shows the characteristics of the selected studies including Gillard et al. (2001) who found Adson’s test to have 72% sensitivity and 53% specificity, with improved predictive value when combined with other maneuvers [10]. Sadeghi-Azandaryani et al. (2009) reported high sensitivity (~92%) for Adson’s test but noted variable specificity, particularly in patients with cervical or first ribs [11]. Plewa et al. (1998) demonstrated false-positive rates of 9-20% in healthy volunteers, reflecting nonspecific vascular changes without pathology [12]. Nord et al. (2008) showed high false positives in carpal tunnel patients (42-45%), indicating poor specificity in non-TOS cohorts [13]. Rayan et al. (1995) and Hixson et al. (2017) both emphasized substantial false positives in normal cohorts and concluded Adson’s test lacks standalone diagnostic accuracy [14,15].

**Table 1:** Characteristics of the Selected Studies TOS: Thoracic Outlet Syndrome; CTA: Computed Tomography Angiography; EAST: Elevated Arm Stress Test; PPV: Positive Predictive Value; FP: False Positive; CTS: Carpal Tunnel Syndrome; QUADAS-2: Quality Assessment of Diagnostic Accuracy Studies-2; PRISMA: Preferred Reporting Items for Systematic Reviews and Meta-Analyses

Authors & Year	Population (P)	Exposure / Condition (I)	Comparator (C)	Outcomes (O)	Pathophysiological Findings	Anatomical Impact	Accuracy of Adson’s Test
Gillard et al., 2001 [10]	48 Patients evaluated for suspected TOS in a vascular clinic	Adson’s test as a provocative maneuver	Imaging/clinical correlation; combination with other maneuvers	Mean sensitivity/specificity of provocative tests 72%/53%; Adson reported the highest PPV among maneuvers	Positional narrowing at interscalene triangle affecting subclavian artery flow	Bony and soft-tissue contributors; cervical rib discussed among causes	Reports PPV ≈ 85%; specificity improved when tests combined.
Sadeghi-Azandaryani et al., 2009 [11]	56 Consecutive patients with clinical TOS	Adson’s test	Surgical/imaging diagnosis and clinical course	EAST sensitivity 98%; Adson sensitivity ~92% among highest; variable specificity	Neurovascular compression with symptom reproduction on positional testing	First-rib/cervical-rib and scalene tightness highlighted as structural substrates	Sensitivity ~92% for Adson; specificity not the highest.
Plewa et al., 1998 [12]	53 Healthy volunteers (asymptomatic)	Adson’s test (A & B variants)	Comparison against normal status	False-positive rates for several maneuvers in normal cohorts	Physiologic pulse variability with neck rotation and inspiration can mimic compression	No structural pathology; demonstrates test’s nonspecific vascular changes	FP 9% (Adson A) and 20% (Adson B),
Nord et al., 2008 [13]	48 Patients with carpal tunnel syndrome and healthy controls	Adson’s test (A & B), Roos, etc.	Neurologically defined CTS and normal cohorts	High false-positive rates: CTS 42% (Adson A) / 45% (Adson B); normal cohorts 9%/20%	Neural compression disorders can yield positive TOS maneuvers without pathology	No cervical rib in most; highlights off-target positives	Confirms low specificity in non-TOS cohorts.
Rayan et al., 1995 [14]	100 normal volunteers (200 upper limbs)	Adson, costoclavicular, hyperabduction	Normal status; vascular/neurologic responses recorded	Prevalence of positive responses to maneuvers in normal cohorts	Maneuvers can provoke vascular/neurologic changes absent disease	No structural anomaly; baseline nonspecific responses	Shows substantial rate of positives in normal cohorts.
Hixson et al., 2017 [15]	Systematic review of clinical tests for TOS	Adson’s test among others	Imaging/clinical diagnoses across included studies	Concluded Adson and Roos should not be used alone (Grade B/C evidence)	Provocative tests reproduce symptoms but lack discriminative validity	Mixed; cervical rib among etiologies considered	Indicates inadequate accuracy standalone.

Risk of Bias Assessment

Table 2 summarizes the risk of bias assessment across the included studies. The study by Gillard et al. (2001) was rated moderate risk using QUADAS-2 due to potential referral bias in patient selection [10]. The study by Sadeghi-Azandaryani et al. (2009) demonstrated low risk, supported by clear surgical confirmation as the reference standard [11]. Studies by Plewa et al. (1998) and Rayan et al. (1995) were judged high risk, as both relied on volunteer populations that introduced artificiality and limited generalizability [12,14]. The study by Nord et al. (2008) carried a moderate risk rating because of possible disease misclassification in a case-control design [13]. Finally, the study by Hixson et al. (2017) was rated moderate risk using the Cochrane tool, reflecting heterogeneity across the systematic review’s included studies [15].

**Table 2:** Risk of Bias Assessment QUADAS-2: Quality Assessment of Diagnostic Accuracy Studies-2 (tool used for assessing risk of bias in diagnostic accuracy studies).

Study	Design	Tool	Risk of Bias Rating	Justification
Gillard et al., 2001 [10]	Prospective	QUADAS-2	Moderate	Referral bias possible
Sadeghi-Azandaryani et al., 2009 [11]	Prospective	QUADAS-2	Low	Clear surgical confirmation
Plewa et al., 1998 [12]	Volunteer study	QUADAS-2	High	Healthy controls only
Nord et al., 2008 [13]	Case-control	QUADAS-2	Moderate	Disease misclassification risk
Rayan et al., 1995 [14]	Volunteer study	QUADAS-2	High	Artificial population
Hixson et al., 2017 [15]	Systematic review	Cochrane	Moderate	Heterogeneity of included studies

Discussion

TOS encompasses a spectrum of neurovascular compression disorders affecting the space bordered by the scalene muscles, first rib, and clavicle. Within this group, the vascular subtype represents a smaller but clinically significant entity, particularly when associated with cervical ribs. These anomalous bony structures narrow the interscalene triangle and costoclavicular space, creating a fixed anatomic predisposition for subclavian artery compression. Patients may develop progressive arterial changes, including intimal hyperplasia, post-stenotic aneurysms, or thromboembolism, all of which can manifest as limb ischemia, claudication, or digital embolic phenomena if not diagnosed early. Recognition of these complications is essential, as untreated vascular TOS may carry significant morbidity compared to its neurogenic counterpart. Clinical evaluation often begins with provocative maneuvers that seek to replicate vascular compression under stress. Among these, Adson’s test remains the most historically cited. By combining head rotation, deep inspiration, and arm extension, the maneuver narrows the scalene triangle and elevates the first rib, thereby accentuating pressure on the subclavian artery.

The presence of a cervical rib further amplifies this effect, making the test particularly relevant in anatomically predisposed individuals. Yet, the maneuver’s physiologic basis is also its weakness; normal alterations in intrathoracic pressure and vascular compliance may mimic compression, resulting in positive responses even in healthy patients. This creates difficulty in distinguishing true pathology from benign hemodynamic variation, explaining its controversial diagnostic role. Diagnostic accuracy rests upon the balance between sensitivity and specificity. The relatively high sensitivity reported by Gillard et al. [10] and Sadeghi-Azandaryani et al. [11] suggests Adson’s test is reliable for detecting hemodynamic compromise in symptomatic patients. However, its low specificity, consistently documented in Plewa et al. [12], Nord et al. [13], and Rayan et al. [14], highlights the risk of overdiagnosis when applied broadly. In clinical practice, this imbalance implies that Adson’s test is more suitable as a screening maneuver that raises suspicion rather than as a confirmatory test. Hixson et al. [15] reinforced this interpretation, concluding from their systematic review that Adson’s test should not be employed in isolation, but may retain value when integrated into a cluster of maneuvers or diagnostic algorithms.

The heterogeneity of study findings reflects not only methodological variability but also the complexity of TOS itself. While cervical ribs represent a clear risk factor, other structural contributors including scalene hypertrophy, fibrous bands, or postural abnormalities, may also generate positive responses. Conversely, neurologic conditions such as carpal tunnel syndrome, as demonstrated by Nord et al. [13], can confound results by producing overlapping symptoms or false vascular findings during testing. This illustrates the broader challenge: provocative maneuvers cannot discriminate between diverse etiologies of upper limb symptoms, nor can they distinguish physiologic from pathologic arterial changes. Thus, while Adson’s test retains physiologic plausibility, its diagnostic validity remains limited without supporting evidence from imaging.

Modern clinical practice has shifted toward imaging-based confirmation as the diagnostic cornerstone. Duplex ultrasonography, computed tomography angiography, and magnetic resonance angiography allow dynamic visualization of subclavian artery compression, mapping the exact site of obstruction and guiding surgical planning [16]. In this context, Adson’s test serves as a non-invasive, rapid bedside screen that can justify further investigation in high-risk patients, particularly those with cervical ribs or suggestive vascular symptoms [17]. Its greatest clinical utility lies in triage identifying patients who require advanced imaging and avoiding unnecessary workup in those with negative or equivocal findings. Nonetheless, the main limitations of the available evidence include heterogeneous study designs, reliance on volunteer cohorts, and older datasets that predate modern imaging standards, reducing generalizability to contemporary clinical pathways.

## Conclusions

Adson’s test remains a historically important clinical maneuver for detecting vascular compromise in thoracic outlet syndrome, especially when a cervical rib is present. Evidence shows it has relatively high sensitivity but poor specificity, leading to frequent false positives in asymptomatic individuals. This limits its role as a standalone diagnostic tool. Its greatest utility lies in screening and triage, guiding the need for confirmatory imaging in high-risk patients. The presence of a cervical rib enhances its predictive value by creating a fixed anatomical predisposition to subclavian artery compression. Modern imaging modalities remain the gold standard for definitive diagnosis, with Adson’s maneuver serving as a supportive adjunct. Future research should focus on refining test combinations and integrating clinical findings with imaging to improve diagnostic accuracy.
